# Polydatin Beneficial Effects in Zebrafish Larvae Undergoing Multiple Stress Types

**DOI:** 10.3390/ijerph18031116

**Published:** 2021-01-27

**Authors:** Andrea Pessina, Mariangela Di Vincenzo, Francesca Maradonna, Francesca Marchegiani, Fabiola Olivieri, Basilio Randazzo, Giorgia Gioacchini, Oliana Carnevali

**Affiliations:** 1 Department of Life and Environmental Sciences, DiSVA, Università Politecnica delle Marche, 60131 Ancona, Italy; a.pessina88@gmail.com (A.P.); m.divincenzo@pm.univpm.it (M.D.V.); f.maradonna@univpm.it (F.M.); b.randazzo@univpm.it (B.R.); giorgia.gioacchini@univpm.it (G.G.); 2 Center of Clinical Pathology and Innovative Therapy, IRCCS INRCA, 60100 Ancona, Italy; f.marchegiani2@inrca.it (F.M.); f.olivieri@univpm.it (F.O.); 3 Department of Clinical and Molecular Sciences, DISCLIMO, Università Politecnica delle Marche, 60100 Ancona, Italy

**Keywords:** *Danio rerio*, polyphenols, immune system, antioxidant, anti-inflammatory, interleukins

## Abstract

Polydatin is a polyphenol, whose beneficial properties, including anti-inflammatory and antioxidant activity, have been largely demonstrated. At the same time, copper has an important role in the correct organism homeostasis and alteration of its concentration can induce oxidative stress. In this study, the efficacy of polydatin to counteract the stress induced by CuSO_4_ exposure or by caudal fin amputation was investigated in zebrafish larvae. The study revealed that polydatin can reduced the stress induced by a 2 h exposure to 10 µM CuSO_4_ by lowering the levels of *il1b* and *cxcl8b.1* and reducing neutrophils migration in the head and along the lateral line. Similarly, polydatin administration reduced the number of neutrophils in the area of fin cut. In addition, polydatin upregulates the expression of *sod1* mRNA and CAT activity, both involved in the antioxidant response. Most of the results obtained in this study support the working hypothesis that polydatin administration can modulate stress response and its action is more effective in mitigating the effects rather than in preventing chemical damages.

## 1. Introduction

Numerous studies have reported that polydatin (PD), a natural glycosylated precursor of resveratrol [1] possesses hepatoprotective and anti-inflammatory properties [2,3]: in mammalian models, its administration protects against cisplatin-induced toxicity [4], attenuates spinal cord injury by inhibiting oxidative stress and microglia apoptosis [5], and contrasts reactive oxygen species (ROS)-mediated extracellular trap formation in case of autoimmune pathologies [3]; in the housefly attenuates cadmium-induced oxidative stress via stimulating of antioxidant system and regulating mitochondrial function [6]; in zebrafish with acute alcoholic liver injuries attenuates hepatic fat accumulation, ameliorates lipid and ethanol metabolism and reduces oxidative stress and DNA damage [7]. Importantly, a number of studies in humans have suggested that polyphenols (PPs), including resveratrol and PD, exert pleiotropic pro-healthy effects modulating inflammaging and lipid/glucose metabolism, thus providing evidence that PP-rich food may provide a sound preventive approach to the most common age-related human diseases [8,9]. Despite this evidence clearly confirms polydatin’s beneficial effects, few data are still available to establish whether its action prevents or mitigates threatening outcomes. Zebrafish, during embryo development, results a suitable model to study PP mechanisms that activate in response to external toxic stimuli [10]. Zebrafish develop a fully functional mature immune system when they are 4 weeks old and cellular inflammatory processes develop because of the immune system activation [11]. During the embryonic/larval stage, the innate immunity plays a pivotal role in the defense against external environmental stimuli [12] and promotes tissue repair [13]. Interleukin-1β (IL-1β), tumor necrosis factor alpha (TNFα), interleukin 6 (IL-6) and interleukin 8 (IL-8) are the main proinflammatory mediators that can steer the immune system cells to the damaged sites [14,15]. Caudal fin incision causes an acute inflammatory process, that triggers a rapid increase of ROS. In damaged cells, ROS act as a paracrine signal and induce the release of endogenous molecules, including the above mentioned cytokines and pro-inflammatory chemokines [16]. The first cells to migrate to the site of infection are neutrophils, which will be later replaced by macrophages, recruited to phagocytize cellular debris and bacterial cells [17]. In this regard, in zebrafish larvae it was observed that after caudal fin amputation, the neutrophil phagocytic capacity is limited, and within 20 min after their activation, neutrophils undergo apoptosis. Six hours after tissue damage, macrophages replace neutrophils and they remain abundant until final tissue repair [18]. Copper (Cu) is a biogenic metal playing numerous functions in organism physiological processes. Its limited intake is a problem; however, doses exceeding the recommended alimentary requirement are problematic and toxicity is soon manifested [19,20]. Most studies on Cu toxicity have shown that the induced organic damage occurs mainly via oxidative stress [21]. The transition between Cu oxidation states can generate hydroxyl radicals and to counteract damage, cells produce molecules with anti-oxidant activity. However, when ROS production exceeds the cellular anti-oxidant capacity, oxidative stress occurs. Prolonged oxidative stress is responsible for several human inflammation diseases (HID) and in extreme cases this can lead to cell death through processes such as apoptosis, necrosis, and autophagy [22,23]. In humans, an excess of Cu has been associated with some serious neurodegenerative diseases, such as Parkinson’s, Huntington’s and Alzheimer’s diseases [24], and its unbalanced homeostasis causes several physiological dysfunctions: anemia, mieoloneuropathy [25], and cognitive problems [26]. Regarding fish, exposure to either Cu nanoparticles or CuSO_4_ induces hepatic and intestinal cytotoxicity in *Epinephelus coioedes* [27,28]; in zebrafish, exposure of embryos to CuSO_4_ causes a delayed hatching, swimming alteration and inhibition of neurogenesis [29], while in larvae, damage to the sensory hair cell population with infiltration of leukocytes to neuromasts was documented [30,31]. Starting from these previous evidences, using zebrafish, which has been largely used to test the effects of the exposure to different nature chemical compounds [32,33], and CuSO_4_ as inflammatory agent, inflammation was induced in larvae and PD was used to counteract Cu toxicity. PD beneficial effects were investigated both before and after CuSO_4_ exposure, in order to gain knowledge, in the first case, of eventual PD protective effects and in the second one, of its capacity to heal Cu toxicity. Response to oxidative stress was analyzed by evaluating the expression of genes codifying for superoxide dismutases (*sod1 and sod2*), and catalase (*cat*) mRNA and related activity [34], all central endogenous anti-oxidant enzymes involved in the first-line defense against ROS [6]. In addition, to get stronger evidence regarding PD anti-inflammatory effects, the amputation of the tail was also performed and the onset of inflammation was checked in the different experimental groups. The results obtained in this study strongly support the well-known beneficial effects of PD and suggest that the timing of administration should be considered to benefit from its anti-inflammatory properties.

## 2. Materials and Methods

### 2.1. Fish Rearing

Zebrafish (*Danio rerio*) were obtained by crossing pathogen-free AB strain broodstock as described in Santangeli et al. [35]. Briefly, adults were maintained in 100 L tanks equipped with mechanical and biological filters under to the following conditions: 28 °C, pH 7.0, and a photoperiod of 12 L/12D. Embryos were obtained by natural mating, gently collected and transferred to a 20 L cylindrical hatchery equipped with ventilation to reduce mortality. After 24 h, embryo viability was checked at the stereomicroscope (Leica Wild M3B, Leica Microsystem, Buccinasco, Italy) and embryos were transferred into trays containing 1.5 mL/embryo E3 medium (5 mM NaCl; 0.17 mM KCl; 0.33 CaCl_2_; 0.3 mM MgSO_4_; pH = 7) and kept at 28 °C until hatching. All procedures involving animals were conducted in line with Italian legislation (D.L. 03/04/14 # 26) on experimental animals and all efforts were made to minimize animal suffering.

### 2.2. Experimental Design

Chemical stress trial: once hatched (Time 0 -T0- = 72 h embryos were divided into 6 experimental groups as described below. Each experimental group was set up in triplicate. Both PD and CuSO_4_ were purchase from Sigma-Aldrich (Darmstadt, Germany) and were diluted in E3 medium. Concentrations of PD (400 µM) and CuSO_4_ (10 µM) were chosen based on previously published papers [36,37]. The time of sampling after CuSO_4_ exposure, was decided on the bases of previous tests as described in Supplementary Materials (Figures S1 and S2). The experiment started at T0, (72 h post fertilization -hpf-, corresponding to hatching) and important time points during the trial are represented by T1 = 75 hpf, T2 = 77 hpf; T3 = 78 hpf. All larvae were sampled at 78 hpf.

1. Control-CTRL- control group reared in E3 medium from T0 to T3
2. CuSO_4_- larvae exposed to 10 µM CuSO_4_ from T1 to T2
3. PD-T0- larvae exposed to 400 µM PD from T0 to T1
4. PD-T2- larvae exposed to 400 µM PD from T2 to T3
5. PD + CuSO_4_- larvae exposed to 400 µM PD from T0 to T1 and to 10 µM CuSO_4_·5H2O from T1 to T2
6. CuSO_4_ + PD- larvae exposed to 10 µM CuSO_4_·5H2O from T1 to T2 and to 400 µM PD from T2 to T3

A schematic representation of the experimental design is shown in Supplementary Materials (Figure S2).

Samples for RealTime-PCR and enzyme activity were store at 80 °C till processed. Samples for myeloperoxidase (MPO) assay were fixed in PFA 4% and stored in PBS at +4 °C till processed.

Mechanical stress trial: to further analyze the role of PD in inflammatory processes, an additional trial was set up, focusing on the effects of PD in fish subjected to caudal fin amputation. Embryos were maintained in E3 medium till hatching, T0 = 72 h. The trial was set up in triplicate.

1. CTRL- reared in E3 medium
2. PD-T0- Larvae exposed to PD from T0 to T1 and then reared in E3 m3dium till T3
3. PD-T2 Larvae exposed to PD from T2 to T3 and sampled
4. TAIL CUT- caudal fin amputation at T1 and reared in E3
5. PD + TAIL CUT- larvae were treated with 400 µM PD from T0 to T1 before caudal fin amputation at T1 and then reared in E3 medium
6. TAIL CUT + PD- caudal fin amputation at T1, transferred in E3 medium till T2 and treated with PD (400 µM) till T3.

Prior to amputations, zebrafish were anesthetized in 15 mg/mL tricaine methanesulfonate (MS-222, Sigma Aldrich, Darmstadt, Germany). Since at T1 caudal fin rays do not present bifurcation [38], fins were cut at 50% of their length.

All experimental fish were sampled at T3 and were used only for the MPO test.

A schematic representation of the experimental design is presented in Supplementary Materials (Figure S3).

### 2.3. Myeloperoxidase (MPO) Staining

MPO activity has been used as a marker for zebrafish neutrophil localization in those areas damaged by exposure to CuSO_4_ and verify PD modulatory action. The staining procedure [39,40] was modified ad hoc for zebrafish. Diaminobenzidine (DAB; Sigma Aldrich -Darmstadt, Germany) used as a dye, reacts with H_2_O_2_ and stains cell in brown. The reaction is catalyzed by myeloperoxidase, an enzyme released by neutrophils and monocytes, with powerful pro-oxidant and pro-inflammatory properties. At T3, 5 larvae for each experimental group, were gently collected and fixed in 4% PFA overnight at 4 °C. After fixation, 3× 10’ PBS 1X washes were performed. The working solution was prepared as follows: 30% Alcohol, Benzidine dihydrochloride, 0.132 M ZnSO_4_· 7H_2_O, Sodium acetate, 3% H_2_O_2_, 1 N NaOH, pH 6 ± 0.05 and was added just before larvae soaking. Larvae were incubated in the working solution for about 5–10 min, and the staining was verified under the microscope. Color development was stopped with a 30-s wash with distilled water. Results were detected using a Zeiss Axio Imager.A2 (Oberkochen, Germany) microscope combined with a color digital camera Axiocam 503 (Zeiss, Oberkochen, Germany).

### 2.4. RNA Extraction and cDNA Synthesis

For each experimental group, 5 replicates of 30 larvae were sampled and stored at −80 °C till analyzed. RNA was extracted using RNAzol RT reagent (Sigma Aldrich, Darmstadt, Germany) following the manufacturer’s instructions. and digested with DNase, to eliminate possible genomic contamination. Final RNA concentration (µg/µL) was determined by NanoPhotmeter P-Class (Implen, München, Germany). RNA quality was determined by agarose gel electrophoresis using Midori Green Advance (NIPPON Genetics Europe, Dueren, Germany) stain. 2 µg of RNA were used to obtain cDNA using the High-Capacity cDNA Reverse Transcription kit (Applied Biosystem, Carlsbad, CA, USA) following the manufacturer’s instructions. Synthesized cDNA was stored at −20 °C.

### 2.5. Real Time PCR

Real time PCR was performed as previously described in [41] using a CFX Connect Real-Time PCR Detection System, Biorad, CA, USA) and a specific protocol for each analyzed gene was applied. Dissociation curve analysis showed a single peak in all cases. Relative quantification of the expression of interleukin 1, beta (*il1b),* chemokine (C-X-C motif) ligand 8b, duplicate 1 *(cxcl8b.1),* interleukin 10 *(il10),* superoxide dismutase 1, soluble *(sod1),* superoxide dismutase 2, mitochondrial *(sod2)* and catalase *(cat)* was performed using ribosomal protein, large, P0 (*rplp0*) and ribosomal protein L13 (*rpl13*) as housekeeping genes to standardize results by removing variations in mRNA. Primer sequence, annealing temperature and GenBank Accession numbers are shown in Supplementary Materials (Table S1). Data were analyzed using iQ5 Optical System version 2.1 (Bio-Rad) including Genex Macro iQ5 Conversion and Genex Macro iQ5 files. Modification of gene expression among the experimental groups is reported as relative mRNA abundance (Arbitrary Units). Primers were used at a final concentration of 10 pmol/mL.

### 2.6. Catalase Activity

Catalase activity was performed as previously described in [34]. Zebrafish samples were prepared as described in [42].

### 2.7. Statistical Analysis

Real time PCR and CAT activity results are presented as means ± standard deviation (SD). The normality of the data was assessed using the Shapiro-Wilk test while the variance was checked using the “var.test” R function. One-way ANOVA non-parametric, followed by the Tukey test as a multiple comparisons test was used to compare mRNA and activity levels among experimental groups.

All statistical analyses were performed using the statistical software package Prism5 (Graphpad Software, Inc., San Diego, CA, USA) and R environment, with significance accepted at *p* < 0.05. Letters indicate statistically significant differences among experimental groups.

## 3. Results

### 3.1. Effect of Polydatin on Neutrophils Migration

**Figure 1:**
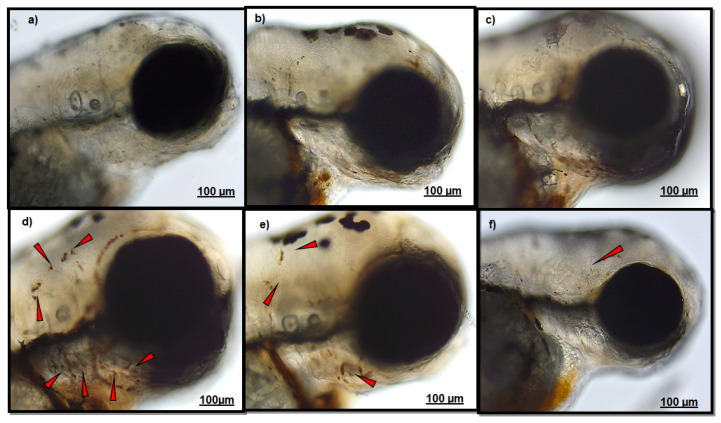
Figure shows representative images of neutrophils localization in the head of: (**a**) CTRL larvae; (**b**) polydatin (PD)-T0 larvae; (**c**) PD-T2 larvae; (**d**) CuSO_4_ larvae; (**e**) PD-CuSO_4_ – larvae; (**f**) CuSO_4_-PD larvae. Red arrows indicate neutrophil localization. Scale bar 100 µM.

**Figure 2:**
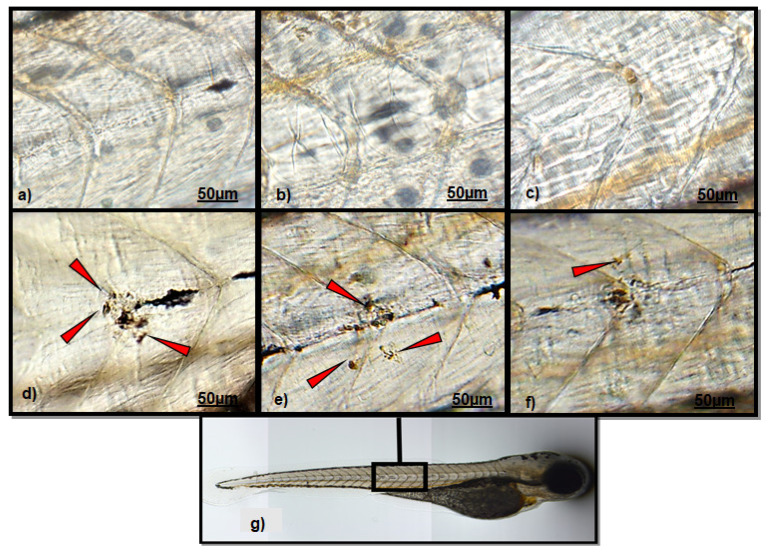
The figure shows representative images of neutrophil localization along the lateral line. (**a**) CTRL larvae; (**b**) PD-T0 larvae; (**c**) PD-T2 larvae; (**d**) CuSO_4_ larvae; (**e**) PD-CuSO_4_- larvae; (**f**) CuSO_4_-PD larvae; (**g**) Box shows the analyzed area. Red arrows indicate neutrophils. Scale bar 50 µM.

An observation analysis of the CuSO_4_-exposed group clearly evidenced a massive localization of neutrophils in both fish head and lateral line (Figure 1d and Figure 2d), respect to those groups not exposed to CuSO_4_ (CTRL (a), PD-T0 (b), and PD-T2 (c)). A lower number of neutrophils were observed in the head of larvae exposed to PD before CuSO_4_ exposure (Figure 1e), respect to larvae exposed to CuSO_4_ alone. A similar neutrophil localization was also detected along the lateral line between larvae exposed to CuSO_4_ alone (Figure 2d) and those exposed to PD before CuSO_4_ exposure (Figure 2e). Conversely, larvae treated with PD after CuSO_4_ exposure showed a lower neutrophil migration in both the head and along the lateral line (Figure 1f and Figure 2f).

**Figure 3:**
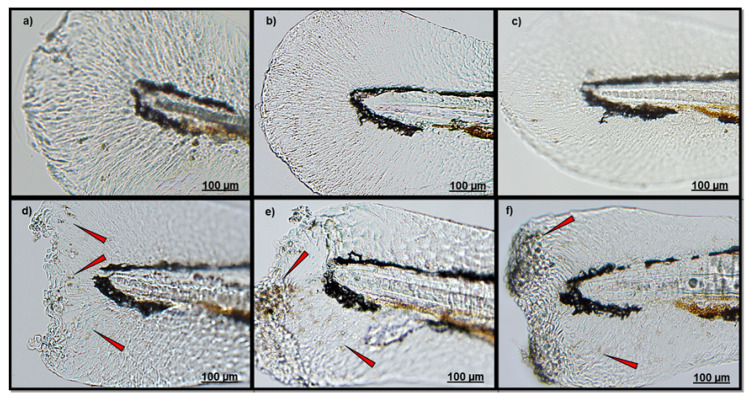
Representative images of neutrophil localization in caudal fin of (**a**) CTRL larvae; (**b**) PD-T0 Larvae; (**c**) PD-T2 Larvae; (**d**) TAIL-CUT larvae; (**e**) PD + TAIL CUT larvae; (**f**) TAIL CUT + PD larvae. All fish were finally sampled at 78 hpf (T3). Red arrows indicate neutrophils. Scale bar 100 μM.

Similarly to CuSO_4_ exposure, tail amputation induced neutrophil migration if compared to that observed in the other groups (Figure 3d). Compared to CTRL (Figure 3a), both larvae treated with PD before (Figure 3e) or after caudal fin amputation (Figure 3f), presented an evident neutrophil localization. 

### 3.2. Effect of Polydatin on Oxidative Stress Pathway

**Figure 4:**
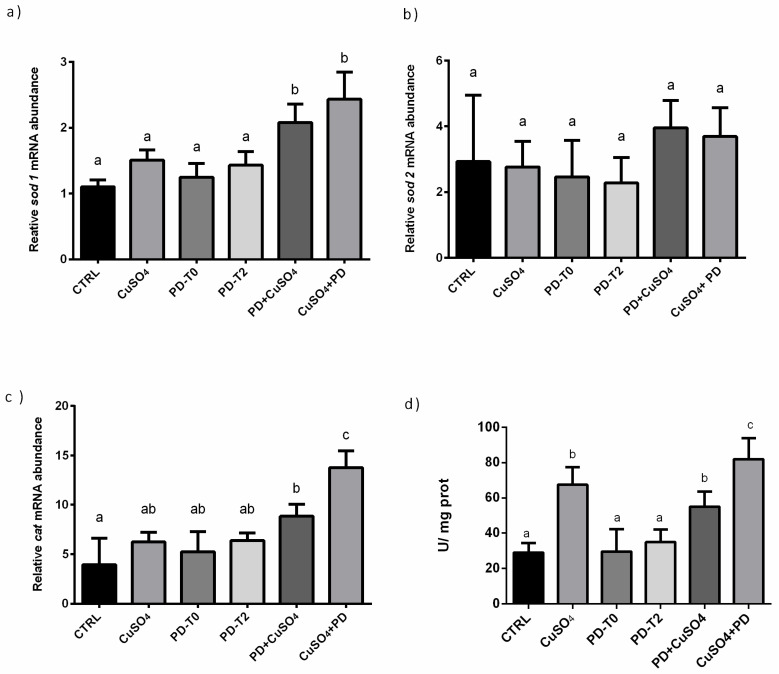
Relative mRNA abundance in larvae groups as described in Materials and Methods—(See Chemical stress trial of (**a**) *sod1*, (**b**) *sod2,* (**c**) *cat* in the different experimental groups as described in Material and Methods section. (**d**) Catalase enzymatic activity. Letters indicate statistically significant differences (*p* < 0.05) among experimental groups. The same letters indicate groups that are not significantly different from each other and vice-versa.

Lack of significant differences of *sod1* expression were observed among CTRL, CuSO_4_, PD-T0 and PD-T2 groups. Conversely, in PD + CuSO_4_ and CuSO_4_ + PD groups, a significantly increase of *sod1* expression levels in respect to all other experimental groups was measured (Figure 4a).

Focusing on *sod2* expression, no differences were found among all experimental groups, despite the fact that in PD + CuSO_4_ and CuSO_4_ + PD groups an increasing trend was observed (Figure 4b).

Similarly, to *sod1,* comparable expression levels of *cat* were observed among CTRL, CuSO_4_ PD-T0, and PD-T2 groups. In PD + CuSO_4_ group, a significantly higher *cat* expression levels respect to CTRL but not respect to CuSO_4_, PD-T0 and PD-T2 was measured. In CuSO_4_ + PD, *cat* expression levels resulted significantly higher respect to all other experimental groups (Figure 4c). Regarding CAT enzymatic activity, levels significantly increased in groups PD + CuSO_4_ and CuSO_4_ + PD, and in larvae exposed to CuSO_4_ in respect to CTRL or groups receiving PD alone, which presented similar values (Figure 4d).

### 3.3. Effect of Polydatin on Inflammation Pathway

**Figure 5:**
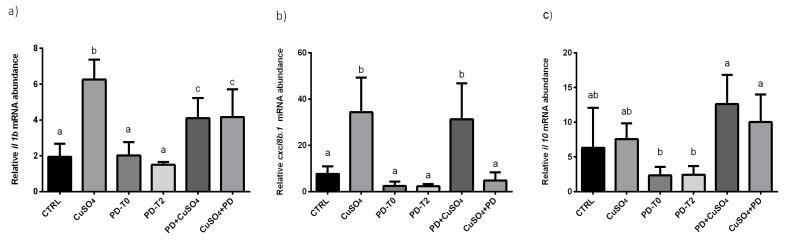
Relative mRNA abundance as described in Materials and Methods—(See Chemical stress trial of (**a**) *il1b*, (**b**) *cxcl8b.1* and (**c**) *il10* in the different experimental groups as described in the Material and Methods section. Letters indicate statistically significant differences (*p* < 0.05) among experimental groups. The same letters indicate groups that are not significantly different from each other and vice-versa.

In CuSO_4_, PD + CuSO_4_ and CuSO_4_ + PD groups, a significant increase of *il1* mRNA levels respect to CTRL was observed. These mRNA levels were significantly higher respect to those measured in groups exposed to PD alone. Similar mRNA levels were measured between PD + CuSO_4_ and CuSO_4_ + PD groups, which resulted significantly lower respect to those observed in CuSO_4_ treated fish (Figure 5a).

Focusing on *cxcl8b.1,* a significant increase was found only in CuSO_4_ and PD + CuSO_4_ groups, respect to all other experimental groups. In CuSO_4_ + PD larvae, mRNA expression was similar to that of CTRL (Figure 5b).

A significant decrease of *il10* expression was detected only in PD-T0 and PD-T2 groups. The treatment with CuSO_4_, alone or administered with PD, did not induce significant changes of mRNA expression (Figure 5c).

## 4. Discussion

Although the beneficial properties of PD have already been described in numerous studies [6,7,43], the data herein obtained provide evidence regarding the best moment for its administration, to maximize its beneficial properties. In fish stresses by CuSO_4_ exposure or by caudal fin cut injured, PD administration induced the expression of anti-oxidant and anti-inflammatory genes and proinflammatory mediator, clearly showing PD beneficial effects as an immune stimulator.

In physiological conditions, inflammatory cells are recruited to the site of wounding or infection by proinflammatory mediators such as hydrogen peroxide, cytokines, and chemokines [44]. Previous studies in zebrafish have shown that, after caudal fin injury, neutrophils localize in the damaged area within 6 h post amputation, before being replaced by macrophages [18]; in case of CuSO_4_ -induced stress, leukocytes migrate close to lateral line neuromast cells for 2–3 h after the stress event [30]. The results herein obtained, integrating the molecular and the myeloperoxidase test data, revealed that PD administration efficacy relies on its ability to mitigate inflammatory outcomes once the stressful event has occurred. Our results evidenced a clear reduction of neutrophils localization in the case of PD treatment, which beneficial effect seems to be higher in the case of exposure to CuSO_4_ rather than in the case of fin injuries.

Once the innate immune response activates, adaptive response-activated cells produce soluble factors needed for lymphocyte recruitment, activation and differentiation. During inflammation and immunity processes, leukocytes and vascular cells interact closely and cytokines play a really important role in this interaction [45]. Oxidative stress is one of the main causes of cellular injuries and the endogenous anti-oxidant defense can prevent ROS-mediated damage [46]. To elucidate the role of PD in the oxidative stress response, the expression of a set of mRNA codifying for proteins with a pivotal role in the organism antioxidant defense, superoxide dismutase (*sod1* and *sod2*) and catalase (*cat*), were investigated [34,47]. SOD is in fact an important endogenous anti-oxidant enzyme that allows a first-line defense against ROS converting O_2_^−^ in O_2_ and H_2_O_2_, while CAT catalyzes the conversion from H_2_O_2_ to O_2_ and H_2_O [48]. Owing to their fast diffusion and versatile biological activities, reactive oxygen species, including hydrogen peroxide (H_2_O_2_), are interesting candidates for wound-to-leukocyte signaling. A study using zebrafish, in fact, showed a sustained rise in H_2_O_2_ concentration at the wound margin, starting 3 min after wounding and peaking at 20 min [49]. In this regard, a treatment with resveratrol significantly reduced hydrogen peroxide-induced oxidative stress in different experimental models [50,51,52] and allowed us to hypothesize about a possible similar action exerted by polydatin in reducing ROS production. Indeed, the molecular analysis revealed that PD treatments allow a greater defense against oxidative stress induced by copper sulphate, with consequent lower activation of the inflammatory response. *Sod1* and *cat* expressions were higher when PD was administered following CuSO_4_ treatment, suggesting a greater beneficial activity as healing rather than as preventive treatment. The difference in expression between the two isoforms of the *sod* gene can be due to the their different cellular localization, *Sod1* being more abundant in cytosol, while *sod2* is more abundant in the mitochondria [53], and suggests that the toxic effects of Cu are mainly neutralized at cytosolic level. In addition, since *Sod1* is a Cu-Zn superoxide dismutase, we can speculate that its upregulation can be firstly due to an increase of cytoplasmatic Cu concentration. In addition, Interleukin 8 gene (*cxcl8b.1*) is involved in neutrophil chemotaxis [54] and its expression increases proportionally to ROS concentration [55]. In this light, our data confirm the benefic effect of PD when administered after the organism is injured, since in this experimental group, mRNA levels were similar to those detected in CTRL larvae and a reduction of neutrophils to the damaged site was shown by MPO assay. Catalase activity strongly supports this evidence; in fact, the highest levels are detected in this group, suggesting that the antioxidant machinery activates to fight against copper-induced ROS production.

Concerning *il1*, previous studies reported that this cytokine can modulate and activate the expression of *il8* [56], and this probably occurs also in our case, since a similar trend of expression was found for these two mRNAs. Differences were only found in the CuSO_4_ + PD group, where the low level of *il8 (cxcl8b.1)* can be caused, as discussed above, by the higher CAT antioxidant activity. Moreover, previous *in vitro* studies, using heat-stressed human keratinocytes [57], demonstrated the beneficial effects of PD on the immune system: IL-6, IL-8, and TNFa gene expression was modulated and HSP70 protein levels increased. Considering that these signals are involved in cytoprotection and cell repair, the increase of *il1* and *cxcl8b.1*, associated with *sod1* levels herein observed, strongly suggests the role of PD in this process also in zebrafish.

Regarding *il10,* its levels were not affected by Cu treatment but were lowered in groups receiving PD alone. The different trends of *il10,* compared to the *il1b* and *cxcl8b.1* ones, is probably due to the different function of these genes. Several studies have so far demonstrated that IL 1b and IL-8 contribute to the defense mechanisms of the host in response to bacterial colonization or invasion [58]. In contrast, the main function of IL-10 seems to be the regulation of the inflammatory response, thereby minimizing damages to the host induced by an excessive response [59]. This suggests that PD, despite its positive role as immune-modulator, acts by a different mode of action in respect to probiotics, which significantly potentiates the host immune response [60] in a tissuespecific manner [61]. PD, indeed, when administered in a healthy organism, does not stimulate their immune system. Conversely, in agreement with Zhao and colleagues [62], in case of stress, PD activates the antioxidant pathway and blocks the ROS-driven inflammasome activation, and as herein observed, induced a lower activation of the immune system by modulating the expression of the pro-inflammatory cytokines *il1* and *cxcl8b.1* and regulating the anti-inflammatory cytokine *il10* mRNA levels.

## 5. Conclusions

In conclusion, as summarized in Table 1, this study further demonstrated that PD can counteract stress by modulating cellular inflammatory and anti-oxidant responses. In particular, here the first insights regarding the timing of PD administration were provided.

## Figures and Tables

**Table 1:** Table summarizing the results obtained analyzing different endpoints in PD-CuSO_4_ and CuSO_4_-PD larvae. Upwards (↑) and downwards (↓) arrows indicate statistically significant changes respect to CuSO_4_ group. Differences of arrow numbers indicate significant differences between PD-CuSO_4_ and CuSO_4_-PD.

			Experimental Groups	
			PD-CuSO_4_	CuSO_4_-PD
Endpoints	MPO Test	Head Localization	↓	↓↓
		Lateral Line	-	↓
	RT-PCR	*sod1*	↑	↑
		*sod2*	-	-
		*cat*	-	↑
		*il 1b*	↓	↓
		*cxcl8b.1*	↑	-
		*il 10*	-	-
	Enzyme Activity	CAT	↑	↑

## Data Availability

Not applicable.

## Supplementary Materials

The following are available online at https://www.mdpi.com/1660-4601/18/3/1116/s1, Figure S1: Representative images showing neutrophil localization in the head, Figure S2: Schematic representation of the Chemical stress trial design, Figure S3: Schematic representation of the mechanical stress trial design, Table S1: Primer List.
