# Supplementary material for: Polydatin Beneficial Effects in Zebrafish Larvae Undergoing Multiple Stress Types
Source: Int J Environ Res Public Health. 2021 Jan 27;18(3):1116. doi: 10.3390/ijerph18031116 (PMC7908490; doi:10.3390/ijerph18031116)
Supplement: Supplementary file 1 [file ijerph-18-01116-s001.pdf]

Supplementary Information Pessina et al.,

Fish exposure to $CuSO_4$

Embryos, obtained by crossing pathogen-free AB adults, were maintained in E3 medium till hatching (T0= 72 hrs) at 28°C. At hatching, larvae were transferred in petri dish and at 75 hpf were exposed for 2 h (till 77 hph) to $CuSO_4$ (10 μM) and then moved back in E3 medium. The activation of the immune response in terms of neutrophil localization was monitored after 1, 3 and 6 hours after the end of the exposure.

As shown in SFig 1, the highest number of neutrophils was visualized in the head (b) within 1 h post exposure and the number decreased after 3 (c) and 6 hours (d). (a) Shows larvae before $CuSO_4$ exposure.

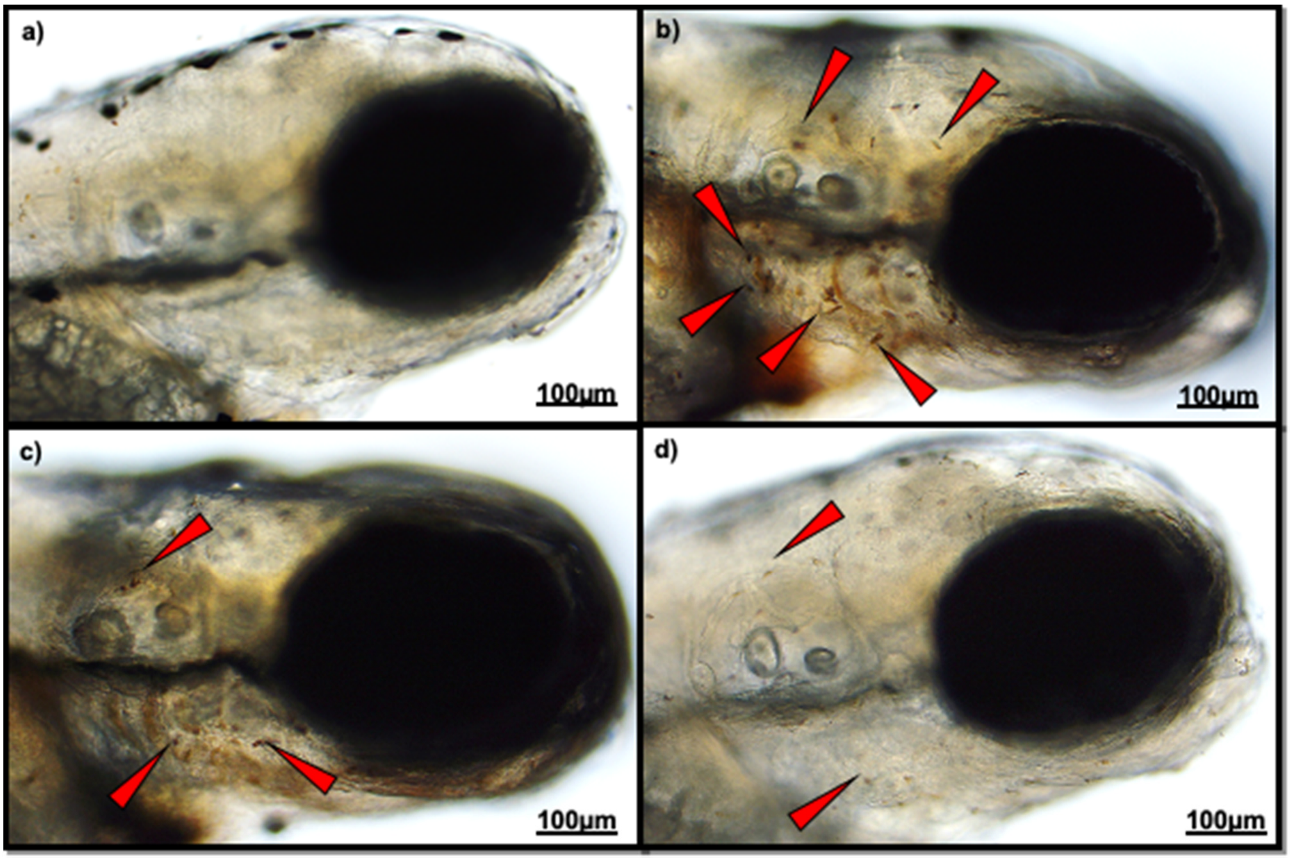

Figure S1. Representative images showing neutrophil localization in the head (a) before $CuSO_4$ exposure and (b) 1 h post $CuSO_4$ exposure, (c) 3 h post $CuSO_4$ exposure and (d) 6 h post $CuSO_4$ exposure. Red arrows indicate neutrophils. Scale bar 100 μM.

Chemical stress trial design

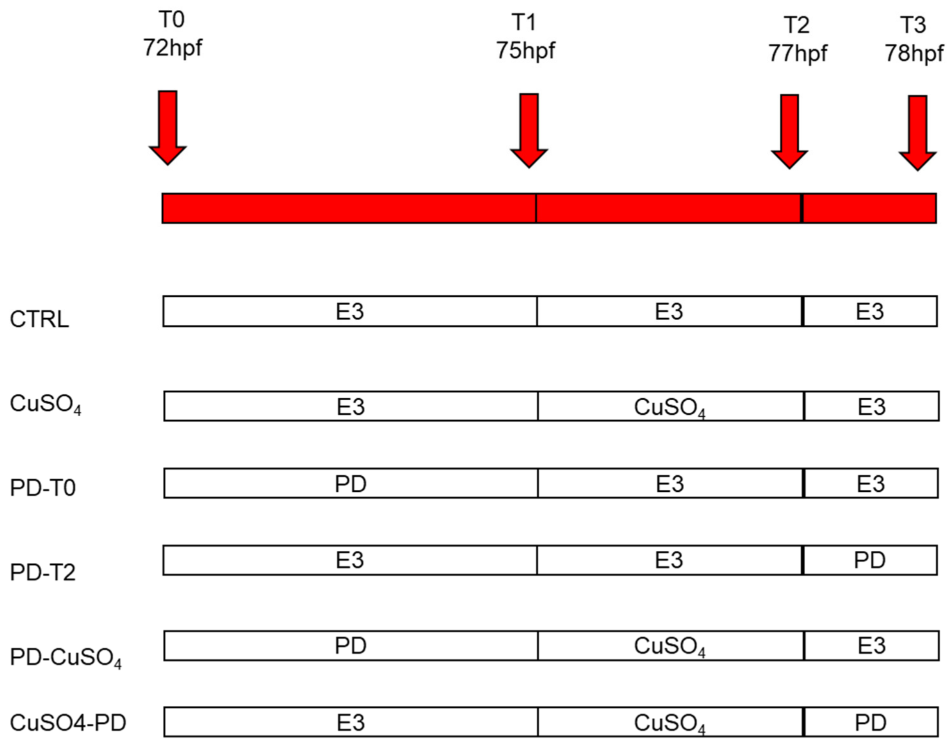

Figure S2. Schematic representation of the **Chemical stress trial** design. Red line shows the different trial time points. White lines show the time of exposure to PD and/or CuSO4 in the experimental groups. E3 = E3 medium, PD = polydatin, T0 = 72hpf, T1 = 75hpf, T2 = 77hpf, T3 = 78hpf

Mechanical stress trial design

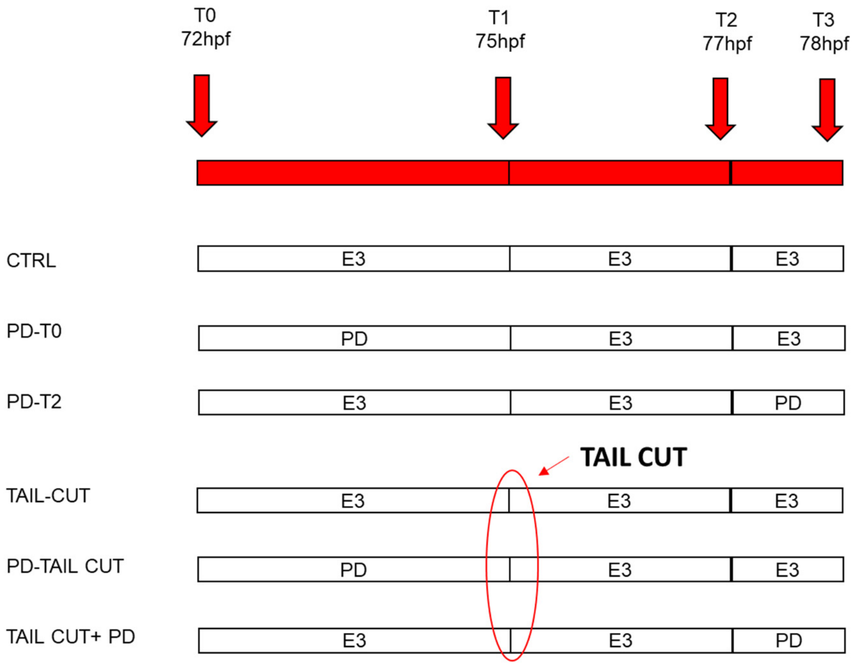

Figure S3. Schematic representation of the **mechanical stress trial** design. Red line shows the different trial time points. White lines show the time of exposure to PD in the experimental groups. Caudal fins were amputated at 75 hpf (T1). E3 = E3 medium, PD = polydatin, T0 = 72hpf, T1 = 75hpf, T2 = 77hpf, T3 = 78 hpf

Table S1. Primer List

Gene	Primer Forward	Primer Reverse	Tm (°C)	GenBank AN
il 1b	GTGGATTGGGGTTTGATGTG	GCTGGGGATGTGGACTTC	54°C	NM_212844.2
cxcl8b.1	ACTCGGACTGAAGGTGACTC	CCACGTCTCGGTAGGATTGAG	58°C	NM_001327985 (1)
il 10	GCTCATTTGTGGAGGGCTTTC	ATTGGGGTTGTGGAGTGCTT	56°C	NM_001020785
sod 1	GTCGTCTGGCTTGTGGAGTG	TGTCAGCGGGCTAGTGCTT	60°C	NM_131294
sod 2	CCGGACTATGTTAAGGCCATCT	ACACTCGGTTGCTCTCTTTTCTCT	60°C	NM_199976 (1)
cat	CCAAGGTCTGGTCCCATAAA	GCACATGGGTCCATCTCTC	60°C	NM_130912 (1)
rplp0	CTGAACATCTCGCCCTTCTC	TAGCCGATCTGCAGACACAC	60°C	NM_131580
rpl 13a	TCTGGAGGACTGTAAGAGGTATGC	AGACGCACAATCTTGAGAGCAG	59°C	NM_198143
